# Social Cognition in Williams Syndrome: Face Tuning

**DOI:** 10.3389/fpsyg.2016.01131

**Published:** 2016-08-02

**Authors:** Marina A. Pavlova, Julie Heiz, Alexander N. Sokolov, Koviljka Barisnikov

**Affiliations:** ^1^Cognitive and Social Neuroscience Unit, Department of Biomedical Magnetic Resonance, Medical School, Eberhard Karls University of Tübingen, TübingenGermany; ^2^Child Clinical Neuropsychology Unit, Department of Psychology, University of Geneva, GenevaSwitzerland; ^3^Department of Women’s Health, Women’s Health Research Institute, University Hospital, Eberhard Karls University of Tübingen, TübingenGermany

**Keywords:** Face-n-Food paradigm, face encoding, face resemblance, social cognition, Williams syndrome, brain mechanisms

## Abstract

Many neurological, neurodevelopmental, neuropsychiatric, and psychosomatic disorders are characterized by impairments in visual social cognition, body language reading, and facial assessment of a social counterpart. Yet a wealth of research indicates that individuals with Williams syndrome exhibit remarkable concern for social stimuli and face fascination. Here individuals with Williams syndrome were presented with a set of Face-n-Food images composed of food ingredients and in different degree resembling a face (slightly bordering on the Giuseppe Arcimboldo style). The primary advantage of these images is that single components do not explicitly trigger face-specific processing, whereas in face images commonly used for investigating face perception (such as photographs or depictions), the mere occurrence of typical cues already implicates face presence. In a spontaneous recognition task, participants were shown a set of images in a predetermined order from the least to most resembling a face. Strikingly, individuals with Williams syndrome exhibited profound deficits in recognition of the Face-n-Food images as a face: they did not report seeing a face on the images, which typically developing controls effortlessly recognized as a face, and gave overall fewer face responses. This suggests atypical face tuning in Williams syndrome. The outcome is discussed in the light of a general pattern of social cognition in Williams syndrome and brain mechanisms underpinning face processing.

## Introduction

Faces convey valuable information for social cognition and non-verbal communication. Many neurological, neurodevelopmental, neuropsychiatric, and psychosomatic disorders are characterized by impairments in visual social cognition, non-verbal communication, body language reading, and facial assessment of a social counterpart (e.g., Pavlova, 2012; Lazar et al., 2014; Pelphrey et al., 2014). A wealth of neuropsychological and brain imaging work suggests that various aspects and stages of face processing are deficient in these disorders (Feuerriegel et al., 2015). Face recognition is reported to be impaired in autistic individuals (Rhodes et al., 2013) and in survivors of preterm birth (Fazzi et al., 2009; Pavlova and Krägeloh-Mann, 2013). Several aspects of face processing are compromised in females with major depressive disorder (MDD; Arrais et al., 2010; Briceño et al., 2015), and with social anxiety (Gilboa-Schechtman and Shachar-Lavie, 2013). Pronounced alterations in face encoding along with brain processing of face affect are reported in eating disorders such as anorexia nervosa and bulimia nervosa (Legenbauer et al., 2008; Pollatos et al., 2008). Yet, in some neurodevelopmental disorders such as Williams–Beuren syndrome (WS), face affect recognition and human body motion perception (which are vital for proper social cognition and interaction) appear to be intact. Moreover, WS individuals exhibit remarkable concern for social stimuli and particular face fascination (e.g., Järvinen et al., 2015).

Williams–Beuren syndrome is a neurogenetic condition [estimated to occur in 1 per 20 000 live births with an assumed equal sex ratio (Bellugi et al., 1999), though more recent estimates have been higher, 1 in 7500 live births (Stromme et al., 2002)], resulting from a homozygous submicroscopic deletion on chromosome 7q11.23 containing the elastin gene. The syndrome is associated with uneven neurocognitive profile with disproportionately severe visual-spatial deficits and relatively spared language abilities (e.g., Bellugi et al., 1988; Majerus et al., 2003). Typically, individuals with WS exhibit specific features such as “elfin” facial appearance, connective tissue malformation, cardiovascular and calcium metabolism problems, and reduced overall brain volume. WS individuals show reduced thalamic and occipital lobe gray matter volumes and reduced gray matter density in regions involved in the visual-spatial system, whereas gray matter volume and density in several areas constituting the social brain and implicated in emotion and face processing (the amygdala, orbital and medial prefrontal cortices, cerebellum, anterior cingulate, fusiform gyrus, and superior temporal gyrus) are preserved or even enlarged (Reiss et al., 2004; Meyer-Lindenberg et al., 2005; Chiang et al., 2007; Campbell et al., 2009; Golarai et al., 2010). Diffusion tensor imaging (DTI) in WS provides evidence for alterations in white matter tracts formations and brain connectivity (Marenco et al., 2007).

Williams–Beuren syndrome individuals possess a hypersocial personality profile, commonly referred to as “cocktail party” style, that is manifested as a friendly, but often exaggeratedly friendly, appetitive drive for social interaction with other people and social closeness, with enhanced emotionality and face processing, and a fondness for music (Jones et al., 2000; Tager-Flusberg et al., 2003; Järvinen et al., 2013). The pattern of the autonomic nervous system response in WS is complex, with increased arousal and lack of habituation to faces (Järvinen et al., 2012). The friendly attitude of those with WS tends to extend to unfamiliar people, leading parents of children with WS to worry about their children’s abnormal tendency to seek out and engage strangers (Gagliardi et al., 2003; Järvinen et al., 2015). Yet individuals with WS do not see all faces as being highly amicable: happy faces are rated as more approachable by individuals with WS (Frigerio et al., 2006). It appears that although individuals with WS discriminate people in terms of approachability, they have difficulty inhibiting their strong compulsion toward social interaction (Frigerio et al., 2006). On the other hand, there is also evidence for poor detection of angry faces in WS (Santos et al., 2010), atypical (reduced) fMRI amygdala response to fear in consort with an increased tendency to approach strangers (Haas et al., 2010), and failure to recruit the amygdala (that is known to be heavily involved in response to fear) during a face discrimination task (Paul et al., 2009).

Despite remarkable face fascination, it remains unclear whether WS individuals are highly tuned to faces: the data on face encoding abilities in WS are controversial, and the outcome appears to depend on the methodology used. WS individuals score in the normal range on standardized face processing tests, e.g., the Benton face test (e.g., Deruelle et al., 2003; Tager-Flusberg et al., 2003; Annaz et al., 2009). Yet it has been suggested that relatively good performance is achieved by atypical underlying processes such as dominant featural face encoding (e.g., Karmiloff-Smith et al., 2003, 2004).

**FIGURE 1:**
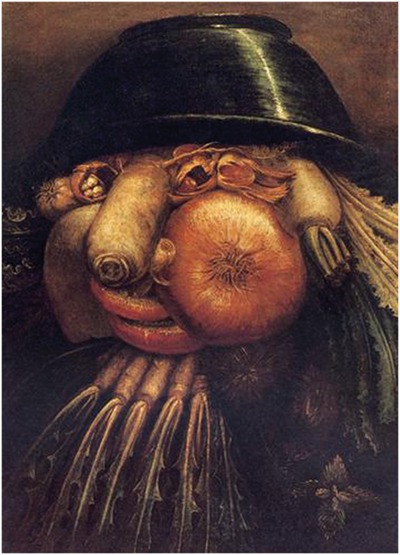
**Example of the Giuseppe Arcimboldo style.** “The Gardner” by Giuseppe Arcimboldo (1526–1593), an Italian painter best known for creating fascinating (often grotesque and allegoric) imaginative portraits composed entirely of fruits, vegetables, plants, tree roots, flowers, and even books and human bodies (http://www.wikiart.org/en/giuseppe-arcimboldo/the-gardner; public domain).

**FIGURE 2:**
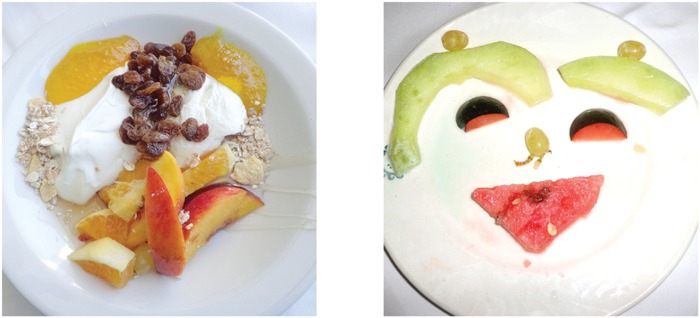
**Examples of images.** The least resembling face **(left panel)** and most resembling face **(right panel)** images from the Face-n-Food task [from Pavlova et al., 2015; the Creative Commons Attribution (CC BY) license].

The present work was aimed at investigation of face tuning in individuals with WS in a recently created Face-n-Food task (Pavlova et al., 2015, 2016). This task consists of a set of food-plate images composed of food ingredients (fruits, vegetables, sausages, etc.) in a manner slightly bordering on the style of Giuseppe Arcimboldo (1526–1593), an Italian painter best known for producing fascinating imaginative portraits composed entirely of fruits, vegetables, plants, and flowers (Pavlova et al., 2015, 2016; **Figures 1** and **2**). One can perceive a Face-n-Food image either as a composition of elements (fruits, vegetables, etc.) or as a Gestalt (a face). The primary advantage of these images is that single components do not explicitly trigger face-specific processing, whereas in face images commonly used for investigating face perception (such as photographs or depictions), the mere occurrence of typical features or cues (such as a nose or mouth) already implicates face presence. This task benefits also from using unfamiliar images that is of special value in clinical settings (Koelkebeck et al., 2015).

Typically developing (TD) adults and children possess an entire bias for seeing faces in Arcimboldo like images. Tuning to faces in the Arcimboldo paintings emerges early in perceptual development: already infants aged 7–8 months prefer the Arcimboldo portraits over the same images presented upside-down (Kobayashi et al., 2012). Patients with prosopagnosia (following right unilateral brain damage) or simultanagnosia are capable of perceiving the Arcimboldo faces (Dalrymple et al., 2007; Busigny et al., 2010).

## Materials and Methods

### Participants

Twenty individuals with WS [10 females, 10 males; who had been diagnosed clinically, and the clinical diagnosis had been confirmed by the genetic diagnosis using fluorescence *in situ* hybridization (FISH) test for elastin (ELN) gene] were enrolled in the study. They were recruited via the Association Suisse du Syndrome de Williams–Beuren, Switzerland, and the Association France Rhône-Alpes, France. Participants were aged 23.3 ± 10.6 years (mean ± SD; age range, 8–44 years). Twenty TD controls pairwise matched with WS individuals for gender and age had been recruited from the local community. Participants were run individually. All of them had normal or corrected-to-normal vision. None had previous experience with such images and tasks. The study was conducted in line with the Declaration of Helsinki and was approved by the local Ethical Committee of the Department of Psychology at the University of Geneva, Switzerland. Informed written consent was obtained from all participants or their parents and care providers. Participation was voluntary, and the data were processed anonymously.

### The Face-n-Food Task

The Face-n-Food task was administered to participants. For this task, a set of ten images was created that were composed of food ingredients (fruits, vegetables, sausages, etc.), and to different degree resembled faces. The images slightly border on the Giuseppe Arcimboldo style (**Figures 1** and **2**). Participants were presented with the set of images, one by one, in the predetermined order from the least to most resembling a face (images 1 to 10). This order was determined in the previous study with TD volunteers (Pavlova et al., 2015). This order had been used since once seen as a face, Face-n-Food images are often processed with a strong face-dominating bias. On each trial, participants had to perform a spontaneous recognition task: they were asked to briefly describe what they saw. Their reports were recorded, and then analyzed by independent experts. For further data processing, the responses were coded as either non-face (0) or face (1) report. No immediate feedback was provided. To avoid time pressure that can potentially cause stress and negative emotional and physiological reactions blocking cognitive processes, there was no time limit on the task. With each participant, the testing procedure lasted for about 20–25 min.

### Neuropsychological Examination

During neuropsychological examination several standardized tests were administered to WS individuals, including Raven’s colored progressive matrices (CPM; Raven et al., 1998), which assesses non-verbal cognitive abilities. CPM scores were in the range from 6 to 29 (17.7 ± 7.62, mean ± SD). In the Visual-Perception subtask (VP) from the Visual-Motor Integration test (VMI, Beery and Beery, 2004; Beery et al., 2010), adapted to French speaking population (Heiz et al., 2014, 2015), participants were first shown a set of progressively complex geometric shapes (in total 27 shapes), and on each item they were asked to choose (point to) the same shape presented among similar shapes (distractors). The test is designed to tap motor-free visual-perceptual skills, and is also used for determination of mental (or developmental) age. VP scores were in the range from 6 to 25 (16.35 ± 5.57, mean ± SD).

## Results

Participants (both TD and WS individuals) either described a food-plate image in terms of food composition (non-face response, 0) or as a face (face response, 1). When an image had been seen as a face, WS individuals similar to TD individuals (see also Pavlova et al., 2015) often gave interpretations in emotional terms (e.g., c’est qqun qui sourit, qui a l’air heureux – it is someone who smiles, who looks happy; un bonhomme comme nous – a man like us). As in the earlier studies with TD young adults (Pavlova et al., 2015, 2016), responses other than face or food (e.g., un oiseau, un papillon – a bird, a butterfly) were given extremely rare, and were coded as non-face reports.

**FIGURE 3:**
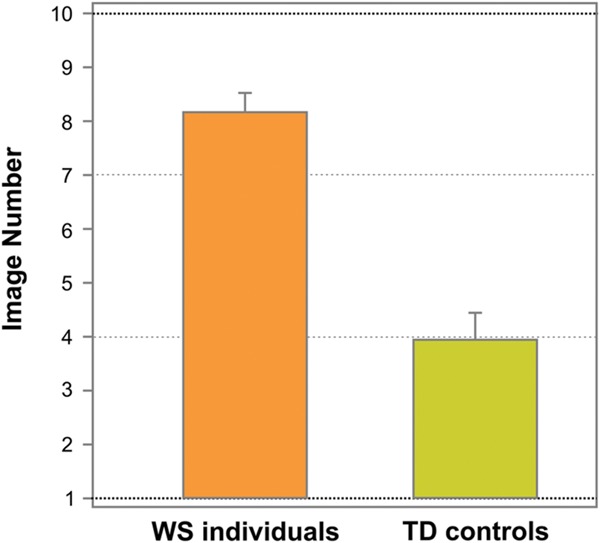
**Tuning to faces.** The average image number, on which resembling a face on the Face-n-Food task (face response) was initially reported, separately for WS and TD individuals. WS individuals experience much more troubles in spontaneous recognition of the images as a face. Vertical bars represent SEM.

**Figure 3** shows the average image number, on which resembling a face (face response) was initially reported on the Face-n-Food task, separately for WS individuals and TD controls. As can be seen from this Figure, WS individuals experience more troubles in spontaneous recognition of the images as a face. TD controls reported seeing a face on average on 3.95 ± 2.24 (mean ± SD) image, whereas WS individuals (17 out of 20) gave the first face response on average only on 8.18 ± 1.47 image. Three out of 20 (15%) WS individuals completely failed on the Face-n-Food task: they did not spontaneously recognize even the most recognizable image number 10 as a face. The difference between WS individuals and TD controls is highly significant [*t*(35) = 6.56, *p* < 0.0001, two-tailed, with an effect size Cohen’s *d* = 2.23].

Once seen as a face, Arcimboldo-like paintings are often processed with a face-dominating bias. However, both WS patients and TD controls gave non-face responses on some subsequent Face-n-Food images. As some perceivers did not report seeing a face on all subsequent images after the initial face report, we performed an additional analysis on the total number of images recognized as a face. The percentage of face responses was 62 ± 19.89 (mean ± SD) for TD controls and only 22 ± 15.1 for WS individuals. The difference in the percentage of face responses between WS individuals and TD controls was highly significant [*t*(38) = 6.94, *p* < 0.0001, two-tailed, with an effect size Cohen’s *d* = 2.27].

**FIGURE 4:**
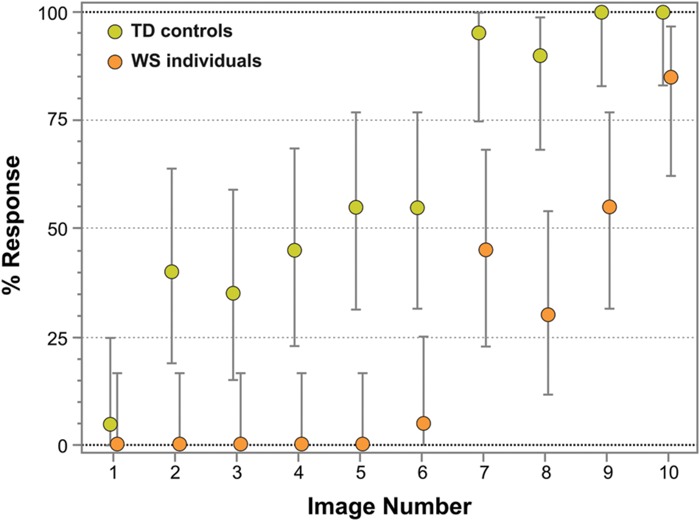
**Percentage of face responses for WS and TD individuals.** The image number reflects its face resemblance (1 – the least recognizable, 10 – the most recognizable as a face). WS individuals not only later report seeing a face and give much fewer face responses, but also later than TD controls reach a ceiling level of performance for their group. Vertical bars represent 95% CI.

**Figure 4** represents the percentage of face responses for each Face-n-Food image for WS individuals and TD controls. As seen from this Figure, WS individuals much later report seeing a face and give overall much fewer face responses: the effect of group (TD controls, WS individuals) is highly significant [χ^2^(1) = 118.21, *p* < 0.0001]. TD controls give almost 50% face responses already from the second image and stay at the same level till the image number 5, whereas WS individuals do not recognize the first five images as a face at all. Starting from the image number 7, TD controls very fast reach the ceiling level of performance. WS individuals much later than controls attain the maximal number of face responses for their group, and still give only 85% face responses even on the most resembling face image number 10. Although face recognition level of WS individuals is much lower, there is no significant interaction between group and image number [χ^2^(9) = 4.91, *p* = 0.842, ns]: the face recognition is just shifted down in WS individuals.

**FIGURE 5:**
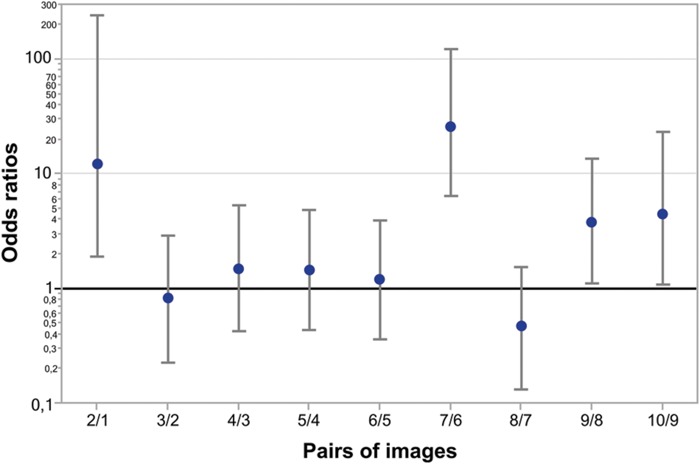
**Odds ratios of face recognition between pairs of Face-n-Food images.** The most prominent leap in face recognition occurs from the image 6 to 7 with an odds ratio of 26 (95% CI 6.5 to 125; *p* < 0.0001). There are non-significant odds ratios between pairs 3/2, 4/3, 5/4, 6/5, and 8/7 that indicates no increase in face recognition. The odds ratios between pairs 2/1, 9/8, and 10/9 are significant showing a hop in face resemblance. Vertical bars represent 95% CI.

**Figure 5** represents odds ratios for all consecutive pairs of Face-n-Food images (independent of group). As seen from this Figure, the most prominent leap in face recognition occurs from the image 6 to 7 with an odds ratio of 26 (95% CI, confidence interval, 6.5 to 125; *p* < 0.0001). The non-significant odds ratios for pairs 3/2, 4/3, 5/4, 6/5, and 8/7 indicate the lack of increase in face recognition. The odds ratios for pairs 2/1, 9/8, and 10/9 are significantly greater than 1; this points to an increase in face recognition. As shown by the likelihood ratio analysis, in TD controls as compared to WS individuals, the odds ratio to give face response to each Face-n-Food image in the set is 46.2 (95% CI, 18 to 158; *p* < 0.0001).

**FIGURE 6:**
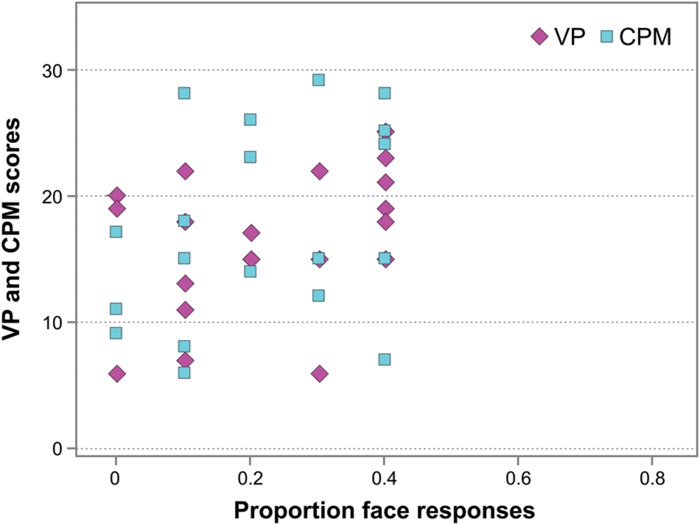
**Face resemblance and the scores on the Visual Perception (VP) test and on Raven’s colored progressive matrices (CPM).** No substantial link occurs between face resemblance (as proportion of face responses) and the scores on the VP and the CPM.

Finally, in WS individuals, no correlation occurred between their performance on the Face-n-Food task (face responses rate) and the scores on the Visual Perception (VP) test from the VMI (Spearman’s rho = 0.352, *p* > 0.05, ns; **Figure 6**), and between the Face-n-Food task performance and mental (or developmental) age as measured by the VP test (Spearman’s rho = 0.379, *p* > 0.05, ns). In addition, there was no link between performance on the Face-n-Food task and the scores on Raven’s CPM (Spearman’s rho = 0.348, *p* > 0.05, ns; **Figure 6**). This indicates that the impaired performance on the Face-n-Food task in the WS group stems from the face tuning deficits rather than from other possible visual-perceptual or non-verbal cognitive disabilities.

## Discussion

The present study was aimed at investigation of face tuning in WS individuals. By using the recently created Face-n-Food task consisting of a set of food-plate images that comprised food ingredients (fruits, vegetables, sausages, etc.; Pavlova et al., 2015, 2016), we investigated tuning to faces in WS and TD individuals. The findings indicate that although WS individuals possess a hypersocial personality profile that is also manifested as a drive for social interaction and particular face fascination, their tuning to faces in the Face-n-Food images is extremely poor. This is rather surprising as faces appear having a special status across different domains of cognitive functioning due to their social relevance. One possible explanation for this outcome is that poor performance on the Face-n-Food task is caused by difficulties of WS individuals in visual feature integration. Indeed, one can perceive a Face-n-Food image either as a composition of elements (fruits, vegetables, etc.) or as a whole or Gestalt (a face). Once seen as a face, the Face-n-Food images are processed with a strong face-dominating bias and, therefore, top-down influences may substantially affect bottom-up visual processing of these images. In line with this, recent findings indicate that original Arcimboldo hidden-face portraits are judged as being more ambiguous by perceivers with local as compared with global perceptual style (Boccia et al., 2014, 2015).

To date, there is no consensus on whether face encoding abilities are preserved in WS. Moreover, this issue is in the focus of heated debate (e.g., Karmiloff-Smith et al., 2003; Annaz et al., 2009; D’Souza et al., 2015). WS individuals score in the normal range on standardized face processing tests, and it is argued that they process faces holistically, similar to TD participants (Deruelle et al., 2003; Tager-Flusberg et al., 2003). Yet, it is also suggested that relatively good performance on face encoding tasks is achieved by atypical underlying processes, in particular the preferential use of featural encoding strategies (e.g., Deruelle et al., 1999; Karmiloff-Smith et al., 2004; Leonard et al., 2011). This is also indicated by a reduced face inversion effect in WS (e.g., Deruelle et al., 1999; Annaz et al., 2009). Developmental studies report atypical developmental trajectories of face processing (Karmiloff-Smith et al., 2004; Annaz et al., 2009; Dimitriou et al., 2014). Infants with WS discriminate between the familiar schematic face (similar to smilies) and (novel) featurally changed faces, but not between the familiar faces and (novel) configurally changed schematic faces, presumably because they pay more attention to the face features than to the whole Gestalt (D’Souza et al., 2015). Yet 5- to 35-month-old toddles with WS process upright, but not inverted, faces holistically, as TD individuals do, that points to their perceptual competence in face encoding (Cashon et al., 2013).

Only a few brain imaging studies investigated brain activation in WS during face encoding. Electroencephalography (EEG) and magnetencephalography (MEG) during processing of upright and inverted faces reveal a number of alterations in WS individuals as compared with TD controls (Mills et al., 2000; Nakamura et al., 2013). WS individuals exhibit less functional magnetic resonance imaging (fMRI) brain activation in the primary and secondary visual cortices (Mobbs et al., 2004), and in early visual areas of the face processing network (Binelli et al., 2016). More extensive brain activation in WS was observed in the right inferior, superior, and medial frontal gyri, anterior cingulate, and several subcortical regions including the anterior thalamus and caudate (Mobbs et al., 2004). The nature of these alterations in brain activation is unclear, and may reflect some compensatory strategies. Fusiform face area (FFA) was found to be two times larger among WS than TD individuals (despite normal levels of face recognition performance on the Benton face recognition test in both groups) that can lead to face recognition proficiency (Golarai et al., 2010). Differences in EEG gamma band oscillations that are thought to underlie visual binding of elements suggest that, although both WS and autistic individuals tend to rely more on featural processing in face recognition, the precise nature of featural processing differs between the two disorders: In autism, apparently normal bursts of gamma activity occur, but they are similar for upright and inverted faces, whereas in WS, no clear gamma peaks were observed for both upright and inverted faces (Grice et al., 2001). The evidence on face encoding in WS remains mixed, with some arguments for typical holistic processing and other arguments for atypical development with a preference for featural encoding (Annaz et al., 2009). Finally, no evidence of exceptional tuning in the brain response to faces (as revealed by visual event-related potentials, ERPs) is recently reported in WS individuals (Key and Dykens, 2015).

For understanding WS, it is of importance to figure out whether face processing has a special status in WS individuals. This is essential also in the light of profound “non-face” visual perceptual deficits. It is worth mentioning that in the present study, face tuning on the Face-n-Food task does not relate to other visual perceptual abilities as measured by the VP of the VMI test (Beery and Beery, 2004) as well as to mental (or developmental) age. No link occurs also between the Face-n-Food task performance and the scores on Raven’s CPM, which assesses non-verbal cognitive abilities. This indicates that the poor performance on this task does not relate to other VP difficulties or possible intellectual disability. In other words, the impaired task performance in the WS group stems from face tuning specifically as opposed to a number of other alternative explanations that can be ruled out here.

Williams-Beuren syndrome individuals are reported to be unhindered in the other social cognitive domains such as visual biological motion processing, which is considered a hallmark of social cognition (Pavlova, 2012). WS children aged 9–18 years and adults perform well on visual biological motion tasks: They can recognize point-light actions (jumping, slipping on a banana) and possess intact ability for direction detection (facing left or right) of a point-light walker moving as if on a treadmill and embedded in a static or dynamic simultaneous mask (Jordan et al., 2002; Reiss et al., 2005). At the same time, children and adults with WS are severely impaired on a 2D form-from-motion task. MEG in a single WS patient aged 20 years indicates that the peak amplitude and latency of the evoked response to point-light biological motion over the right occipitotemporal cortex do not substantially differ (is within two standard deviations) from those of TD controls (Hirai et al., 2009). To date, there is a lack of consensus in regard to the ability of WS individuals to detect coherent motion. There are contradictory data indicating that this ability is either impaired (Atkinson et al., 2006) or preserved (Nakamura et al., 2002; Reiss et al., 2005). As spotlighted earlier (Pavlova, 2012), this is an important issue because in the light of deficient ability for other kinds of motion processing, the selective sparing of biological motion processing (similar to face processing) would point to its special status.

The present work suggests a limited ability of WS individuals for seeing faces in the Face-n-Food images, and may be considered a first step toward putting the Face-n-Food task into clinical setting. Taking into account appetitive social drive and face fascination in WS individuals, this outcome appears arresting. One possible explanation for this outcome is that the Face-n-Food test is much more sensitive to preferences for the featural face encoding strategy in WS individuals. As mentioned earlier (Pavlova et al., 2015), methodological issues [such as the nature of stimuli: real (photos and movies), depicted, or arty faces; different stages of face encoding addressed; task demands that may be non-specific to face processing itself] may be of potential value for the outcome of face processing studies.

Further step in elaborating the Face-n-Food task in WS would be recording the brain activity. Much closer look at specific topographic patterns and temporal dynamics of the neural circuitry underpinning facial processing (with hubs in the FFA and posterior superior temporal sulcus, STS, which are considered pivots of the social brain) can add essential information on typical and atypical processing of the Face-n-Food images. For uncovering face processing in the social brain, one may take an advantage of ultra-high field fMRI providing for much higher sensitivity and spatial resolution along with concurrent EEG recording to simultaneously obtain precise spatial and temporal information.

## Conclusion

In a nutshell, the outcome of the study indicates that WS individuals exhibit profound deficits in seeing faces in the Face-n-Food images represented by a composition of food ingredients in a manner bordering on the Giuseppe Arcimboldo style. WS individuals did not report seeing a face on the images, which TD individuals effortlessly recognize as a face, and gave overall substantially fewer face responses. This suggests atypical face tuning in WS despite their hypersocial personality profile that is manifested as a drive for social interaction and particular face fascination. The precise nature of this abnormality including the brain mechanisms underlying face encoding, remains to be clarified. The Face-n-Food task may serve as a valuable tool for uncovering impairments in visual face processing in neurological, neurodevelopmental, and neuropsychiatric disorders such as autistic spectrum disorders, schizophrenia or depression.

## Author Contributions

Conceived and designed the experiments: MAP. Performed the experiments: JH and KB. Analyzed the data: MAP, JH, ANS, and KB. Contributed reagents/materials/analysis tools: MAP and KB. Wrote the paper: MAP, ANS, and KB. Supervised the whole project: MAP.

## Conflict of Interest Statement

The authors declare that the research was conducted in the absence of any commercial or financial relationships that could be construed as a potential conflict of interest.
